# The role of islet autoantigen-specific T cells in the onset and treatment of type 1 diabetes mellitus

**DOI:** 10.3389/fimmu.2024.1462384

**Published:** 2024-09-24

**Authors:** Mengmeng Yue, Xianzhen He, Xinwen Min, Handong Yang, Hao Xu, Wenwen Wu, Jixin Zhong, Aihua Mei, Jun Chen

**Affiliations:** ^1^ Sinopharm Dongfeng General Hospital (Hubei Clinical Research Center of Hypertension), School of Basic Medical Sciences, Hubei University of Medicine, Shiyan, China; ^2^ Children’s Medical Center, Renmin Hospital, Hubei University of Medicine, Shiyan, Hubei, China; ^3^ School of Public Health, Hubei University of Medicine, Shiyan, Hubei, China; ^4^ Department of Rheumatology and Immunology, Tongji Hospital, Huazhong University of Science and Technology, Wuhan, Hubei, China; ^5^ Shiyan Key Laboratory of Virology, Hubei University of Medicine, Shiyan, China

**Keywords:** type 1 diabetes mellitus, islet autoantigen, antigen-specific T cells, T cell phenotype, immunotherapy

## Abstract

Type 1 diabetes mellitus (T1DM), a complex chronic disease with an intricate etiology and pathogenesis, involves the recognition of self-antigens by pancreatic islet autoantigen-specific T cells and plays crucial roles in both early- and late-stage destruction of beta cells, thus impacting disease progression. Antigen-specific T cells regulate and execute immune responses by recognizing particular antigens, playing broad roles in the treatment of various diseases. Immunotherapy targeting antigen-specific T cells holds promising potential as a targeted treatment approach. This review outlines the pathogenesis of diabetes, emphasizing the pivotal role of pancreatic islet autoantigen-specific T cells in the progression and treatment of T1DM. Exploring this avenue in research holds promise for identifying novel therapeutic targets for effectively managing diabetes.

## 1 Introduction

Type 1 diabetes mellitus (T1DM) is one of the most prevalent and severe chronic diseases, with complications that can endanger life, cause disability, and shorten lifespan (1). T1DM is a complex autoimmune disorder characterized by a combination of genomic, epigenomic, and environmental factors affecting adaptive and innate immune cell populations, ultimately leading to pathological chronic inflammation of the pancreas (2). These factors influence the pathophysiology of autoimmune T1DM, the speed of disease progression, and the degree of pancreatic beta cell dysfunction.

T cells play a central role in the pathogenesis of T1DM, with both CD4(+) and CD8(+) T cells involved in its development (3, 4). CD8(+) T cells are the predominant T cell population and the most abundant inflammatory cell type in insulitis. Using HLA multimers, investigators demonstrated the antigen specificity of autoreactive CD8(+) T cells in insulitis lesions of T1DM donors (5). The diversity of the antigen repertoire of infiltrating CD8(+) T cells increases with the progression of the disease (5). Antigen-specific T cells regulate and execute immune responses by recognizing specific antigens. Traditional broad-spectrum immunosuppressive agents have serious side effects. For the treatment of autoimmune T1DM, targeted and specific immunotherapy must be developed to rebuild immune tolerance or eliminate the beta cell-specific immune response, thereby preserving the function of the beta cells population. Immunotherapy based on antigen-specific T cells holds significant potential as a targeted therapeutic option, with the promise to prevent or reverse T1DM (6–8).

This article discusses the pathogenesis of T1DM. Unlike other relevant literature reviews, this article emphasizes the crucial roles of various autoantigen-specific T cells from the perspective of specific immune cells in the onset of T1DM. Recent research on autoreactive antigen-specific T cells and their clinical applications is systematically reviewed.

## 2 Pathogenesis of type I diabetes mellitus

T1DM is an immune-mediated type of diabetes characterized by organ-specific autoimmune destruction that isnotably mediated by antigen-specific T cells targeting pancreatic beta cells (9). In the study of the pathogenesis of T1DM, T1DM results from the combined influence of genetic susceptibility, immune responses, and environmental factors, all of which are closely associated with the onset of T1DM (10).

### 2.1 Genetic susceptibility

While most T1DM patients lack a family history, genetic susceptibility plays a crucial role in the autoimmunity and destruction of beta cells. The human leukocyte antigen (HLA) region is currently considered the most potent genetic determinant, contributing to 40–50% of the genetic risk of T1DM. In genome-wide studies, many other genetic variations, including the insulin gene, are associated with T1DM, some of which are linked to suspected environmental risk factors, indicating a complex polygenic inheritance pattern (11, 12). The mechanisms of susceptibility genes in T1DM may involve their respective roles in antigen presentation, beta cell autoimmunity, immune tolerance, and self-reactive T cell responses. Environmental susceptibility factors also increase the risk of developing T1DM. From an epigenetic perspective, the pathological mechanisms underlying T1DM may involve DNA methylation, histone modifications, microRNAs, and molecular mimicry. These mechanisms may affect the immune system’s response to beta cells by regulating gene expression (13).

### 2.2 Environmental factors and nutritional factors

Although T1DM is an autoimmune disease, environmental factors, including infections, immune stimuli, early-life environments, lifestyle, etc., can influence the expression of susceptibility genes, accelerating or delaying the progression of the disease.

Exposure to specific microorganisms is indeed a crucial factor (14). The interaction between the gut microbiota and the immune system is indeed a key factor in the pathogenesis of T1DM. Insulin dysfunction and the mechanism of T1DM onset may be associated with changes in the composition of the gut bacteria. Higher levels of microbial diversity, beneficial microbes, and production of microbial metabolitest are utilized as protective agents against the onset of T1DM (15, 16). Indeed, most research on the impact of the gut microbiota on the pathogenesis of T1DM has been conducted in animal models. More human studies are needed to substantiate this concept.

Evidence also suggests that nutrition plays an important role in the development of diabetes (14). High-dose calcitriol (an active metabolite of vitamin D3) has been shown to reverse the onset of chronic insulitis and diabetes in NOD mice, delaying the progression of the disease (17). Vitamin D regulates insulin secretion in the pancreas and insulin sensitivity in multiple peripheral metabolic organs through the vitamin D receptor. It improves glucose homeostasis by increasing insulin secretion, reducing inflammation, decreasing autoimmunity, preserving the beta cell mass, and increasing insulin sensitivity. Conversely, vitamin D deficiency is associated with an increased incidence of T1DM (18). The potential protective effect of vitamin D on the development of T1DM may be attributed to its immunomodulatory properties. These properties help to suppress chronic pancreatic inflammation. Additionally, Yukiko Kagohashi et al. reported that the ratio of essential fatty acids in the maternal diet during pregnancy and lactation influences the onset of insulin production in offspring. In studies on NOD mice, they strongly prevented T1DM in offspring (19). To some extent, the influence of nutritional factors on the development of diabetes remains controversial (14, 20). More evidence is needed to elucidate the relationships between nutritional elements and the development of T1DM.

In summary, the pathogenesis of T1DM is a complex process involving the interactions of genetic, immune, and environmental factors. Understanding these mechanisms is crucial for the prevention, treatment, and management of T1DM. Overall, the pathogenesis of type 1 diabetes is a complex process involving the interactions of genetic, immune, and environmental factors. A thorough understanding of these mechanisms helps us better comprehend the development of the disease and provides more effective approaches for prevention and treatment.

### 2.3 Activation of the immune system

The pathogenesis of T1DM involves intricate interactions within the immune system and its regulatory mechanisms, with extensive documentation of dysregulation of both cellular and humoral immune responses, particularly cellular immunity (11, 21).

The immune system mistakenly identifies pancreatic beta cells as foreign entities, leading to their impairment and destruction. This process results in compromised insulin secretion and dysregulated blood sugar control, ultimately triggering the onset and progression of T1DM (22). Antigen-specific immune cells, such as CD4(+) T cells and CD8(+) T cells, along with other immune cells such as natural killer cells and macrophages, are activated and directed to attack pancreatic beta cells. This autoimmune attack results in a reduction in the number of pancreatic beta cells and impaired insulin secretion.

Chronic inflammation can sustainably activate the innate immune system, exerting long-term detrimental effects on insulin secretion and function. Additionally, it can lead to complications related to diabetes involving both the macrovascular and microvascular systems (23). Inflammation within the pancreatic lymph nodes leads to a decrease in the expression of peripheral tissue antigens, resulting in the generation of new pancreatic islet antigens. These antigens may not be effectively tolerated by T cells. This phenomenon promotes the escape and activation of autoreactive T cells, thereby accelerating the onset of T1DM (24).

Some studies highlight the critical role of the intestinal immune system in regulating insulin-specific responses triggered by dietary insulin (25). Notably, insulin-specific humoral and T cell immunity can be initiated in early infancy through exposure to insulin in the diet (26). In children with T1DM, abnormalities in the intestinal immune system are evident and are characterized by increased immune activation and increased permeability. These factors collectively impair the regulation of dietary insulin tolerance (27).

Moreover, compelling evidence suggests a potential association between rotavirus infection and the exacerbation or onset of pancreatic autoimmunity in genetically susceptible children (28). As elucidated by Burke et al., this association is closely linked to immune responses against infection (29). Additionally, enteroviruses have been detected in the pancreas of diabetic patients. Viral antigens and receptors have been identified in beta cells, surrounding macrophages, and T lymphocytes in pancreatic tissue from patients with fulminant T1DM (30). Subsequently, the immune response to viral infection accelerates the destruction of beta cells within the pancreas.

Immune system activation has a significant effect on the progression of T1DM, directly affecting the function and survival of pancreatic beta cells and indirectly increasing the risk of complications. Therefore, controlling immune system activation to prevent autoimmune damage to islets is crucial for managing T1DM and preventing complications.

## 3 The role of Islet autoantigen-specific T cells in diabetes

**Figure 1:**
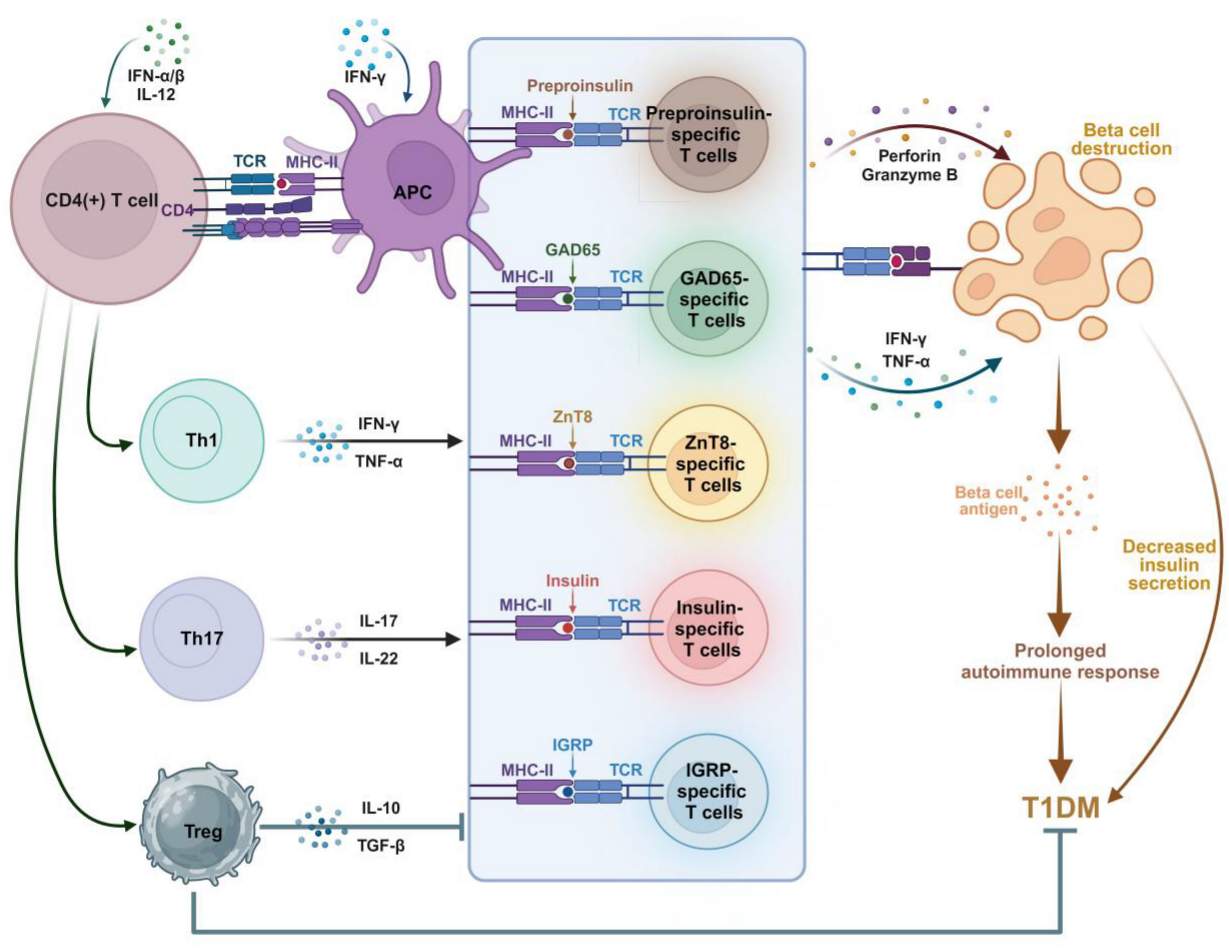
Involvement of islet autoantigen-specific T cells in the pathogenesis of T1DM. Autoantigens are processed by antigen-presenting cells (APCs) and presented to naive CD4(+) T cells via HLA class II MHC molecules. Activated CD4(+) T cells shift to (i) Th1 phenotype, releasing IFN-γ and TNF-α cytokines; (ii) Th17 phenotype, releasing IL-17, IL-22 and other inflammatory cytokines; And (iii) Tregs, which secret anti-inflammatory IL-10 and TGF-β. Various autoantigen-specific CD8(+) T cells release perforin, granzyme B, IFN-γ, and TNF-α in response to autoantigens. These cytotoxic factors destroy beta cells, resulting in reduced insulin release. Meanwhile, IL-10 and TGF-β released by Tregs inhibit the activity of autoantigen-specific T cells, leading to the suppression of T1DM. Islet inflammation prolongs the autoimmune response and accelerates the progression of T1DM. Figure created with BioRender (biorender.com).

CD8(+) T cells specific for multiple autoantigens are detected in the islets of patients with long-term disease (5). The specificity of the pancreatic islet antigen is essential for the accumulation of T cells in the pancreatic islets (31). T1DM is typically accompanied by elevated levels of autoantibodies. The appearance of insulin autoantibodies represents a crucial step in the development of beta cell autoimmunity (32, 33). The production of these autoantibodies is closely associated with the activation of T cells in the body, which play a crucial role in initiating or altering the insulin-specific autoimmune response in the pathogenesis of T1DM (32, 33). In the prediabetic phase, various autoreactive T cells respond to pancreatic islet cell antigens (**Table 1**) (**Figure 1**). Pancreatic islet autoantigen-specific T cells serve as markers of the autoimmune destruction of cells (34). In studies using animal models, pancreatic islet-specific T cells have been shown to identify self-antigens and play crucial roles in both early- and late-stage beta cell destruction. Beta cell-specific CD8(+) T cells lead to the destruction of pancreatic beta cells, resulting in insulin deficiency and the loss of glucose homeostasis (35). Notably, ectopic expression of CD80 in beta cells has been shown to accelerate the onset of insulin-dependent diabetes mediated by beta cell-specific CD8(+) T cells, whereas diabetes mediated by CD4(+) T cells remains unaffected. This observation underscores the antigenic interaction between beta cells and the aforementioned CD8(+) T cells (36). Compared with healthy controls, patients newly diagnosed T1DM exhibit antigen-driven expansion of the compartment of beta cell-specific CD8(+) T cells. Consequently, oligoclonal populations of beta cell-specific CD8(+) T cells may exist within the memory repertoire of healthy individuals without evidence of disease activity (37).

**Table 1:** Characteristics and roles of different types of islet autoantigen-specific T cells.

Autoantigen-specific T cells	Identify epitopes/proteins	How to play a pathogenic role	Role in T1DM	Possible therapeutic implications	References
**Preproinsulin-specific T cells**	signal peptide; B-chain; C-peptide; A-chain; (PP_I15-24_; PPI_2–10_)	Escape central and peripheral tolerance; Express IFN-γ; Induce beta cell death via cytotoxic degranulation;	Attack the islets, leading to destruction of beta cells;	Reducing the frequency of PPI-specific T cells and limiting their proliferative potential can delay the progression of T1DM;	(38, 39)
G**AD65-specific T cells**	GAD_114-123_;	Present in the early stages of the disease; Shift immune balance towards inflammatory phenotype; Attack pancreatic beta cells;	Trigger inflammation and enhance autoimmune response;	Aid in early diagnosis and intervention; Improve disease management and patient prognosis; Contribute to the development of vaccine-based targeted prevention strategies for T1DM;	(40)
**ZnT8-specific T cells**	ZnT8_186–194_ epitope; ZnT8_153–161_ epitope;	Present in the early stages of the disease; Involved in beta cell destruction;	Participate in diabetes onset under conditions of pancreatic immune impairment;	ZnT8-specific immunotherapy could be a substitute approach for treating T1DM;	(41)
**Insulin-specific T cells**	B-chain amino acid sequence B:_9-23_; insulin B_15–23_;	Prediabetes can be detected; Islet infiltration; Trigger antibody-dependent cytotoxicity against beta cells; Insulin epitope mutations lead to escape of highly pathogenic T cells;	Involved in islet infiltration during prediabetes; Promotes destruction of beta cells; Activates local endothelium to assist penetration of other T cells into the islets;	Understanding mechanisms of escaping negative selection in the thymus helps in developing preventive treatments;	(42, 43)
**IGRP-specific T cells**	IGRP_206-214_; IGRP_228–236_;	Cluster in the islets early; Secrete IFN-γ and granzyme B; The number increases with the progression of islet inflammation and age;	Attacking pancreatic beta cells, inducing destructive insulitis; A crucial component of early islet infiltration.	Monitoring the activity levels of IGRP-specific T cells to assess disease status and predict disease progression, guiding personalized T cell therapy; Lowering cell affinity contributes to disease protection;	(44–46)

### 3.1 Preproinsulin-specific T cells

Proinsulin (PPI) is the precursor of insulin. Pancreatic beta cells process and present proinsulin to themselves, rendering themselves targets for CD8(+) T cell-mediated killing (47). In T1DM, PPI-specific CD8(+) T cells are activated to evade central and peripheral tolerance and attack the pancreatic islets, leading to the destruction of beta cells. Among beta cell-related antigens, PPI plays a crucial role in the pathogenesis of T1DM (48, 49). Krishnamurthy et al. reported that autoreactive T cells targeting the self-antigen proinsulin are positioned upstream of the response to islet-specific glucose-6-phosphatase catalytic subunit-related protein (IGRP), indicating that the pathogenic autoimmune response to proinsulin subsequently spreads to other antigens (50). PPI-specific CD8(+) T lymphocytes are present in the pancreas and peripheral circulation of nondiabetic individuals. As diabetes progresses, these cells accumulate in and around the pancreatic islets (51). Clones of PPI-specific CD8(+) T cells derived from diabetic patients exhibit a proinflammatory phenotype and are capable of killing surrogate beta cells and human HLA-A24(+) islet cells *in vitro*. The number of PPI-specific CD8(+) T cells is increased in recently diagnosed patients (47). Clones of PPI-specific CD8(+) T cells primarily rely on cytotoxic degranulation to induce beta cell death. Moreover, variations in the killing capacity of PPI-specific CD8(+) T cells are not due to inherent differences in the cells themselves but are mediated by differences in the peptide-HLA ligand signal strength (52). PPI-specific CD8(+) T cells can express IFN-γ and promote diabetes. IFN-γ is a pleiotropic cytokine that may influence immune and autoimmune responses by supporting the homing of activated T cells. A deficiency in IFN-γ delays the development of autoimmune diabetes (53, 54). Subsets of PPI-specific CD8(+) T cells are relatively confined to the central memory, and their low frequency and limited proliferative potential are associated with the slow progression of adult-onset T1DM (55). Unlike that of polyclonal Tregs, the frequency of PPI-specific Tregs varies among different subtypes of T1DM. The distinct memory-like phenotype of PPI-specific Tregs is attributed to chronic antigen presentation rather than being dependent on the age at disease onset (56).

### 3.2 GAD65-specific T cells

Glutamic acid decarboxylase of 65 kDa (GAD65) is an enzyme involved in the synthesis of gamma-aminobutyric acid, which is an important neurotransmitter in the central nervous system (57). GAD65 is also one of the primary pancreatic antigens targeted by autoreactive T cells in patients with T1DM (58). The immune system mistakenly identifies GAD65 as a foreign antigen and mounts an immune response against it. In most cases, the appearance of GAD65-specific autoantibodies occurs before the destruction of beta cells (59). Self-antibodies against GAD65 are detected in early-diagnosed T1DM patients at a frequency of approximately 80% (60). Research by Nehenuo Chuzhoet al. (61) suggests that GAD65 peptides may be presented to CD4(+) T cells by HLA-DR3 molecules, HLA-DQ2 molecules, or both (GAD65 peptides containing amino acids 288–308 may be more readily presented to CD4(+) T cells by HLA-DR3 molecules), inducing the expression of the cytokines IFN-γ and IL-17 in the CD4(+) T cells of T1DM patients and potentially shifting patients from immune balance to an inflammatory phenotype. GAD65-specific T cells are among the first cells to enter inflamed islets and are widely present in the peripheral blood of T1DM patients (62, 63), playing a crucial role in the pathogenesis of this disease (64). Activation of GAD65-specific T cells leads to autoimmune attack on pancreatic beta cells.

Research on GAD65-specific T cells is crucial for understanding the immune mechanisms of T1DM and developing targeted treatments. Moreover, given the early infiltration of GAD65-specific T cells into pancreatic islets during inflammation, detecting and monitoring these cells in individuals at risk of T1DM may assist in early diagnosis and intervention, ultimately enhancing disease management and patient prognosis, and may contribute to the development of vaccine-based targeted prevention strategies for T1DM.

### 3.3 ZnT8-specific T cells

ZnT8 has transporter 8 (ZnT8) is a dimeric transmembrane protein regarded as a regulator of the zinc concentration within beta cells. It facilitates the transport of zinc from the cytoplasm to secretory vesicles, playing a crucial role in zinc accumulation and insulin secretion in beta cells (65, 66). Deficiency or downregulation of ZnT8 can affect insulin biosynthesis, release, and beta cell function through both direct and/or indirect mechanisms (67).

ZnT8 has cellular or tissue specificity and is expressed primarily in pancreatic islets. ZnT8 has been identified as a novel target autoantigen in patients with type 1 diabetes. It possesses intrinsic immunogenicity and readily binds to the Fab2 and Fc regions of autoantibodies. Upon binding to immune effector cells, it triggers antibody-dependent cellular cytotoxicity against beta cells (68–70). Reportedly, ZnT8-specific CD8(+) T cells have been detected in the serum of the majority of individuals in prediabetic and diabetic states (69, 71). Studies have also confirmed that individuals with T1DM have a greater presence of ZnT8-specific CD8(+) T cells in the pancreas than do patients with type 2 diabetes or nondiabetic individuals (72). Sefina Arif reported that, compared with healthy adults, T1DM patients have a higher prevalence of ZnT8-specific CD4(+) T helper (Th) 1 cells. Conversely, in healthy controls, Th2 cells and IL-10-producing cells predominate among ZnT8-specific CD4(+) T cells. Additionally, proinsulin-specific CD4(+) T cells secrete IL-10 rather than IFN-γ (73). Daisuke Chujo’s research further extended these findings, indicating a significant increase in ZnT8-specific T cell subsets in T1DM patients compared with healthy adults (74). However, some studies suggest that ZnT8 is only a minor diabetogenic antigen (75) and that it participates in diabetes development under conditions of pancreatic immune damage. The immune response of CD4(+) T cells to ZnT8 is weakly pathogenic. Nevertheless, ZnT8-specific T cells play a crucial role in the pathogenesis of T1DM to some extent. Investigating the functions and regulatory mechanisms of these immune cells contributes to a deeper understanding of the pathophysiology of T1DM and provides important clues for the development of new treatment strategies.

### 3.4 Insulin-specific T cells

Insulin serves as one of the earliest self-antigens targeted by autoreactive CD4(+) and CD8(+) T cells, leading to immune-mediated destruction of beta cells in patients with T1DM (76). Insulin-specific T cells are the predominant component of pancreatic islet infiltration during the prediabetic stage in NOD mice and contribute to the destruction of beta cells during the development of diabetes in NOD mice (77, 78). These cells rapidly induce diabetes in NOD mice and noninflammatory mice (42). Maria Bettini and Marissa A. Scavuzzo, among others, presented evidence that insulin epitope mutations lead to the escape of highly pathogenic T cells. In the absence of homologous antigens, the antigen reactivity, clonality, and pathogenicity of insulin-specific T cells increase (79). Insulin-specific T cells (80, 81) and islet antigen-specific CD8(+) T cells (40) are rarely detected in healthy individuals (38). Compared with GAD65- or ZnT8-specific T cells, the presence of pancreatic islet-specific CD8(+) T cells may be more restricted to individuals with T1DM.

### 3.5 IGRP-specific T cells

IGRP (islet-specific glucose-6-phosphatase catalytic subunit-related protein)is the major islet glucose-6-phosphatase anchored in the endoplasmic reticulum of pancreatic beta cells via nine transmembrane domains (82) and is a critical component controlling glucose substrate cycling and energy metabolism in pancreatic beta cells. Research has shown that IGRP is an antigen for CD8(+) T cells (83). IGRP-specific CD8(+) T cells play a unique role in the pathogenesis of T1DM, secrete IFN-γ and granzyme B upon antigen-specific activation, and participate in the immune attack on pancreatic beta cells, inducing destructive insulitis (84).

In patients with acute-onset T1DM, the frequency of IGRP-specific CD8(+) T cells is significantly elevated (45, 85). Moreover, in peripheral lymphoid tissues, the number of IGRP-specific T cells that induce destructive insulitis correlates with the progression of islet inflammation and increases with age (effector-memory T cells develop in islets and are a marker of islet pathology in individuals with type 1 diabetes). Given this characteristic, evaluating disease status and predicting disease progression by monitoring the activity level of IGRP-specific T cells in the blood appear feasible to guide individualized treatment plan designs and thus offer a future direction for diagnosing and monitoring disease progression in T1DM patients.

### 3.6 Other

In the pancreas, chromogranin-A (ChgA) is a beta cell secretory granule protein (86) and one of the self-antigens in NOD mice and T1DM patients (87). Vasostatin-1 is a naturally processed fragment of ChgA (one of the antigen targets of pathogenic CD4(+) T cells). Nikoopour E. et al. found that vasostatin-1-specific T cells constitute a significant portion of the pancreatic islet-infiltrating T cells in diabetic NOD mice, contributing to the onset of type 1 diabetes in NOD mice (88).

As T1DM progresses, the pancreas harbors an increasing number of pancreatic islet antigen-specific CD8(+) T cells with a rich memory phenotype (89). Autoantigen-specific CD8+ T cells in the islet-draining lymph nodes of NOD mice exhibit a strong self-renewal ability and can rapidly differentiate into effector cells and destroy beta cells (35). Autoantigen-specific CD8+ T cells (confirmed by class I MHC tetramers) display stem-like epigenetic features and are able to maintain activity after prolonged exposure to autoantigens (90). These cells are also present in the bloodstream and pancreas of healthy individuals, but they are not enriched within the pancreatic islets (51). In healthy individuals, autoreactive CD8(+) T cells can survey the pancreas and eliminate malfunctioning cells. In diseased states, increased availability of pancreatic antigens or enhanced T cell activation may augment this killing and trigger disease. Therefore, the phenotype and function of peripheral blood CD8(+) T cells may reflect the health status of beta cells and the activation state of T cells (91). In summary, these studies support the role of pancreatic islet antigen-specific T cells in the progression of this disease.

Whether multiple autoantigen-specific T cells in T1DM develop independently or propagate from one to another requires further investigation. The identification and in-depth studies of the activity and function of these T cells can uncover the breadth and complexity of the autoimmune response and aid in pinpointing therapeutic targets. However, despite advances in understanding the pathogenic role of autoantigen-specific T cells, little is known about the origin and mechanisms of autoreactive autoimmune T cells, as well as their mechanisms of escaping negative selection within the thymus.

## 4 Current status of research and clinical application of antigen-specific T cells as therapeutic targets for diabetes

### 4.1 Disease prediction and surveillance

Islet-specific autoantibodies can be present in the body for weeks or even up to 20 years before clinical onset (14, 92). Monitoring various autoantigens and specific serum autoantibodies can predict future diabetes by assessing ongoing beta cell autoimmunity and islet pathology in high-risk populations (93, 94). Research has indicated that the quantity of peripheral blood islet-specific CD8(+) T cells may serve as a predictive biomarker for T1DM (95). Monitoring beta cell-specific T cells can track diabetes induction responses in high-risk populations or diabetic patients (96). The number of IGRP-specific T cells in peripheral lymphoid tissue correlates with the progression of islet inflammation and increases with age (97). Moreover, after the diagnosis of diabetes, a stable number of IGRP-specific T cells corresponds to a memory phenotype (84, 97), serving as one of the indicators for monitoring diabetes. Insulin-specific CD4(+) T cell responses can serve as novel markers for assessing residual endogenous beta cell function and predicting better 2-year disease outcomes (98).

### 4.2 Disease diagnosis

The autoimmune status of T1DM depends on islet autoantibodies. For example, GADA (glutamic acid decarboxylase antibody) is an easily detectable biomarker that can be detected months before clinical presentation and can serve as a biomarker for diabetes in adults (99, 100). Research has shown that ELISA combined with antibody testing can improve the diagnostic sensitivity for autoimmune diabetes (101). Multiple islet antigens combined with CD4(+)-ELISA-ACDC and direct testing can provide cellular immune diagnostic value for patients with T1DM (102).

By studying islet autoantigen-specific T cells, individuals in preclinical or high-risk groups can be identified. Early diagnosis aids in implementing interventions promptly to delay or prevent the development of T1DM.

### 4.3 Immunotherapy

Current treatment strategies for T1DM patients rely primarily on exogenous insulin injection or insulin pumps, which can save lives but not cure the disease. Although insulin replacement is beneficial for improving glycemic control, the biological function of endogenous insulin is limited, increasing the risk of hypoglycemia in T1DM patients (103).

The US Food and Drug Administration (FDA) approved teplizumab (an anti-CD3 monoclonal antibody) in November 2022 to delay or prevent T1DM. In early clinical trials, a teplizumab immunotherapy intervention has shown benefits in preserving beta cell function (104) and significantly reducing the risk of developing type 1 diabetes (105). The approval of teplizumab is widely considered important, marking a new approach in biologics for treating T1DM that surpasses traditional insulin replacement therapy to address potential autoimmune factors (106). Teplizumab has been engineered to have a non-activating Fc region, that inhibit the activity of autoreactive T lymphocytes (107). The beneficial effects of teplizumab treatment may result in part from partial or transient T cells exhaustion. Exhausted T cells are characterized by loss of effector function (cytokine production and proliferation); expression of multiple inhibitory receptors; differential connectivity of transcription factors; low metabolic activity (108–110), and dependence on continuous presence of antigen. Thus, islet autoreactivity of CD8(+) effector T cells was reduced (110). In clinical trials, it was observed that CD3 antibody could increase Tregs and decrease the ratio of CD4(+) to CD8(+) T cells. It can delay or prevent the progression of T1DM by restoring self-tolerance and reducing the autoimmune destruction of islet beta cells (111, 112). The success of teplizumab provides inspiration for the development of more personalized and targeted treatment strategies. The development of personalized immunotherapy strategies based on patients’ immune characteristics and genetic backgrounds can effectively treat and manage T1DM.

Inducing T cell-specific tolerance to self-antigens is a therapeutic goal for T1DM, but it may suppress autoimmunity in populations at high risk of T1DM (113, 114). Antigen-specific approaches can target inflammatory lymphocytes, induce apoptosis, or prevent their migration to the pancreas. A lack of costimulation, Tregs expansion, and bystander suppression may be mechanisms by which antigen-specific immunotherapy (ASI) regulates pathogenic T cells (115). Identifying T cells and their specific antigenic epitopes may provide immunotherapeutic targets for personalized treatment (116). Research has shown that autoimmune diabetes can be prevented using immunodominant T cells epitopes (117). A ZnT8-specific monoclonal antibody (mAb43) binds with high affinity to ZnT8 on beta cell surfaces, demonstrating pancreatic specificity *in vivo* and thereby enhancing the safety of targeted therapy. It provides sustained protection against autoimmune diabetes in NOD mice. Treatment with mAb43 may represent a viable long-term solution for preventing or reversing newly diagnosed T1DM (118). In summary, specific antigenic epitopes may serve as immunotherapeutic targets to improve beta cell dysfunction in T1DM patients, providing new avenues for treating T1DM (69).

Tregs are recognized as inhibitors of autoimmune responses (119). Antigen-specific Tregs can effectively reverse established autoimmune responses in T1DM patients, suppress antigen-specific T cell responses to multiple antigens, alleviate clinical symptoms, and be employed in immunotherapy for T1DM patients (120, 121). Tissue-specific immune tolerance can be achieved through beta cell-specific Tregs without compromising general immune function (122). Insulin-specific Tregs are uniformly distributed in lymphoid tissues but are relatively rare. A study by Neda Đedović and colleagues introduces a new, reproducible experimental approach for enriching and expanding insulin-specific Tregs, offering potential for cellular therapy for autoimmune diseases (123).

Insulin-specific Th1 cells isolated from NOD mice can migrate to the pancreas, preventing spontaneous and adoptive transfer-induced diabetes. They seem to act locally by releasing transforming growth factor-β and/or other factors that inhibit the homing and/or proliferation of immune cells within the pancreas. These findings may provide insights into and suggest mechanisms for the protective effects of insulin therapy on diabetes (124).

In individuals with T1DM, ASI represents an effective and safe treatment approach to induce immune tolerance. While single antigen therapy shows promise in animal models (69), it has not yet achieved prevention or reversal in human clinical trials. Thus optimizing these immune therapies and possibly employ combination treatments are urgently needed to further improve treatment efficacy. The variability in islet autoantigen-specific T cell activity and specificity among individuals suggests the potential for personalized treatment measures, such as developing targeted immunomodulatory therapies to lower or prevent the occurrence of T1DM.

### 4.4 Disease prevention

Research on pancreatic autoantigen-specific T cells contributes to understanding the pathogenesis of T1DM, laying the foundation for the development of preventive strategies. Intervening with or modulating the activity of these antigen-specific T cells may prevent or delay the onset of T1DM. The disruption of natural immune mechanisms leads to autoimmune diseases, but antigen-specific therapies that enhance biological mechanisms provide promising safe and effective treatments for preventing T1DM (125). Antigen-specific therapy requires two components: self-antigens and safe methods to induce T cell tolerance to these antigens (126).

Minor adjustments in response to pancreatic antigen-specific T cells may be sufficient to prevent the clinical onset of T1DM in genetically susceptible individuals (127). Mutation of insulin CD8(+) T cell epitopes can prevent the onset of diabetes in NOD mice. Modified peptide ligands effectively induce antigen-specific T cell apoptosis, atrophy, or immune response transfer for antigen-specific prevention of T1DM (128). Following autoimmunity, antigen-specific T cells initially differentiate into antigen-specific memory cells, and the immune response gradually spreads to multiple antigens (126).

Another critical area involves developing and testing new preventive or therapeutic “vaccines” to induce immune tolerance in beta cell through the targeting of effector molecules and/or the modulation of the specificity of self-reactive CD8(+) T cells (129). A plasmid DNA vaccine encoding mouse insulinoma antigen II can decrease the incidence of diabetes in T1DM mouse models. Antigen-specific plasmid DNA therapy represents a viable strategy for preventing the progression of T1DM (130). A detailed understanding of specific targets for T cell memory responses has become a primary tool in conceptualizing and developing preventive or therapeutic vaccines (116, 131).

Using vaccination based on self-antigens to prevent harmful immune responses against oneself is considered the best strategy for addressing autoimmune diseases. This method depends on eliminating or rendering pathogenic T cells inactive or promoting the generation of beneficial Tregs (132). Compared with polyclonal Tregs, antigen-specific Tregs are more effective at inhibiting pathogenic immune responses (133). Recent studies have indicated that the generation of antigen-specific Tregs can suppress memory T cells (126), but inducing tolerance at this stage remains challenging. Preclinical studies are necessary to determine the additional “assistance” needed to induce tolerance in memory T cells and to develop effective treatment strategies for individuals with existing autoimmunity.

After preventive treatment, evaluating the effectiveness and long-term effects of preventive strategies can be achieved by monitoring the activity of pancreatic autoantigen-specific T cells. This approach helps in adjusting and improving preventive strategies promptly to increase their effectiveness. Research on insulin immunity is crucial in predicting diseases.

### 4.5 Treatment of islet allograft rejection and anti-host disease

Islet transplantation is an effective treatment option for T1DM, especially for patients who are unable to maintain good blood sugar control through other means. Studying pancreatic autoantigen-specific T cells is essential for the treatment and management of islet transplantation in T1DM patients to increase transplant success rates, lower rejection risks, and optimize personalized treatment strategies.

After islet transplantation, patients may face the risk of graft rejection of transplanted islet cells, where immune system attack is a primary mechanism. Studies indicate that pancreatic antigen-specific T cells are involved in the dysfunction of transplanted islets. Islet autoantigen-specific T cells precede chronic graft dysfunction in islet transplant recipients, and these T cells are correlated with chronic graft dysfunction (134).

The induction of antigen-specific T-cell tolerance in islet transplant patients promotes the treatment of various immune diseases and aids in preventing allograft rejection and graft-versus-host disease (135). On the other hand, inducing the tolerance of T cells specific to autoantigens may suppress autoimmunity in patients receiving islet transplantation or regenerative therapy (113), and more research is needed to explore the possibility of achieving this goal without affecting general immune function.

## 5 Summary and prospects

In conclusion, establishing autoantigen-specific tolerance is an effective strategy for treating immune system diseases. Meaningful and detectable biomarkers in antigen-specific therapy have not yet been determined yet, and questions persist regarding the optimal molecular form, dosage, and route of antigen administration. By advancing cellular technologies to elucidate the subtle differences in phenotype and function determined by specificity, researchers can gain deeper insights into autoimmune processes in T1DM, which is crucial for developing novel treatments for T1DM. The regulation of antigen-specific T-cell cytotoxicity is a potential target for disease treatment control, but further investigation into the possibility of blocking self-reactive CD8(+) T cell function without compromising general immune function is needed. Additionally, further research is needed to confirm whether these antigen-specific T cells are primary drivers initiating autoimmune processes and their impacts on the development of T1DM. Furthermore, most preventive or therapeutic immune therapies have been successfully applied only in animal models and not yet in humans. More research is needed to assess and improve their safety, stability, and efficacy.
